# Triage, treatment and transfer of patients with stroke in emergency department trial (the T^3^ Trial): a cluster randomised trial protocol

**DOI:** 10.1186/s13012-016-0503-6

**Published:** 2016-10-18

**Authors:** Sandy Middleton, Chris Levi, Simeon Dale, N. Wah Cheung, Elizabeth McInnes, Julie Considine, Catherine D’Este, Dominique A. Cadilhac, Jeremy Grimshaw, Richard Gerraty, Louise Craig, Verena Schadewaldt, Patrick McElduff, Mark Fitzgerald, Clare Quinn, Greg Cadigan, Sonia Denisenko, Mark Longworth, Jeanette Ward, On behalf of the T3 Trialist Collaborators, Chris May, Rohan Grimley, Richard Paolini, Rosemary Phillips, Enna Salema, Janne Pitkin, Toni Sheridan

**Affiliations:** 1 Nursing Research Institute, St Vincent’s Health Australia (Sydney) and Australian Catholic University, Executive Suite, Level 5 DeLacy Building, St Vincent’s Hospital, Victoria Road, Darlinghurst, 2010 New South Wales Australia; 2 John Hunter Hospital, Newcastle, Australia; 3 Centre for Translational Neuroscience and Mental Health, University of Newcastle/Hunter Medical Research Institute, Newcastle, Australia; 4 Centre for Diabetes and Endocrinology Research, Westmead Hospital and University of Sydney, Westmead, Sydney, New South Wales Australia; 5 Faculty of Health, Eastern Health - Deakin University Nursing and Midwifery Research Centre School of Nursing and Midwifery, Burwood, Victoria 3125 Australia; 6 National Centre for Epidemiology and Population Health (NCEPH), Australian National University, Canberra, Australian Capital Territory Australia; 7 Stroke and Ageing Research, School of Clinical Sciences at Monash Health, Monash University, Clayton, Melbourne, Victoria Australia; 8 Florey Institute of Neuroscience and Mental Health, University of Melbourne, Parkville, Victoria Australia; 9 Clinical Epidemiology Program, Ottawa Health Research Institute, 1053 Carling Avenue, Administration Building, Room 2-017, Ottawa, Ontario K1Y 4E9 Canada; 10 Department of Medicine, University of Ottawa, 451 Smyth Road, Ottawa, Ontario K1H 8M5 Canada; 11 Department of Medicine, Monash University, Melbourne, Australia; 12 Neurosciences Clinical Institute, Epworth Hospital, Richmond, Victoria 3121 Australia; 13 School of Medicine and Public Health, University of Newcastle, Newcastle, New South Wales 2300 Australia; 14 Alfred Hospital, Melbourne, Victoria 3004 Australia; 15 Department of Surgery, Central Clinical School, Monash University, Melbourne, Australia; 16 Faculty of Science, Engineering and Technology, Swinburne University of Technology, Melbourne, Australia; 17 Speech Pathology Department, Prince of Wales Hospital, High St, Randwick, New South Wales 2031 Australia; 18 Statewide Stroke Clinical Network, Brisbane, 4000 Australia; 19 Department of Health Victoria, Victorian Stroke Clinical Network, Melbourne, Victoria 3000 Australia; 20 Stroke Services NSW, NSW Agency for Clinical Innovation, Chatswood, New South Wales Australia; 21 School of Epidemiology, Public Health and Preventive Medicine (SEPHPM), University of Ottawa, 451 Smyth Road, Ottawa, Ontario K1H 8M5 Canada; 22 Nulungu Research Institute, University of Notre Dame Australia, Broome, Western Australia Australia

**Keywords:** Cluster randomised trial, Stroke, Nurse-led, Fever, Hyperglycaemia, Dysphagia, Thrombolysis, Theoretical domains framework, Implementation science, Care bundle

## Abstract

**Background:**

Internationally recognised evidence-based guidelines recommend appropriate triage of patients with stroke in emergency departments (EDs), administration of tissue plasminogen activator (tPA), and proactive management of fever, hyperglycaemia and swallowing before prompt transfer to a stroke unit to maximise outcomes. We aim to evaluate the effectiveness in EDs of a theory-informed, nurse-initiated, intervention to improve multidisciplinary triage, treatment and transfer (T^3^) of patients with acute stroke to improve 90-day death and dependency. Organisational and contextual factors associated with intervention uptake also will be evaluated.

**Methods:**

This prospective, multicentre, parallel group, cluster randomised trial with blinded outcome assessment will be conducted in EDs of hospitals with stroke units in three Australian states and one territory. EDs will be randomised 1:1 within strata defined by state and tPA volume to receive either the T^3^ intervention or no additional support (control EDs). Our T^3^ intervention comprises an evidence-based care bundle targeting: (1) triage: routine assignment of patients with suspected stroke to Australian Triage Scale category 1 or 2; (2) treatment: screening for tPA eligibility and administration of tPA where applicable; instigation of protocols for management of fever, hyperglycaemia and swallowing; and (3) transfer: prompt admission to the stroke unit. We will use implementation science behaviour change methods informed by the Theoretical Domains Framework [1, 2] consisting of (i) workshops to determine barriers and local solutions; (ii) mixed interactive and didactic education; (iii) local clinical opinion leaders; and (iv) reminders in the form of email, telephone and site visits. Our primary outcome measure is 90 days post-admission death or dependency (modified Rankin Scale >2). Secondary outcomes are health status (SF-36), functional dependency (Barthel Index), quality of life (EQ-5D); and quality of care outcomes, namely, monitoring and management practices for thrombolysis, fever, hyperglycaemia, swallowing and prompt transfer. Outcomes will be assessed at the patient level. A separate process evaluation will examine contextual factors to successful intervention uptake. At the time of publication, EDs have been randomised and the intervention is being implemented.

**Discussion:**

This theoretically informed intervention is aimed at addressing important gaps in care to maximise 90-day health outcomes for patients with stroke.

**Trial registration:**

Australian and New Zealand Clinical Trials Registry ACTRN12614000939695. Registered 2 September 2014.

**Electronic supplementary material:**

The online version of this article (doi:10.1186/s13012-016-0503-6) contains supplementary material, which is available to authorized users.

## Background

International clinical guidelines recommend early management of stroke on arrival to the emergency department (ED) in order to improve patient outcomes [3–5]. Key elements of stroke care applicable to EDs are appropriate *triage*; *treatment* by administration of tissue plasminogen activator (tPA) to eligible patients and management of fever, hyperglycaemia and swallowing; followed by prompt *transfer* to an acute stroke unit. Data available at the time of our T^3^ (triage, treatment and transfer) Trial commencement demonstrated variable practices. With regard to triage, allocation of an Australasian Triage Scale (ATS) category 1 (to be seen immediately) or category 2 (to be seen within 10 min) is recommended for patients presenting to EDs with signs or symptoms of acute stroke [6]. However, these targets are not always met; an analysis of Victorian ambulance data demonstrated that 30 % of patients with stroke were not allocated an ATS category of 1 or 2 [7]. Inappropriate triage allocation resulting in delays in assessment and diagnosis also may have a flow-on adverse effect on provision of thrombolysis to patients who may benefit and create delays in implementation of other elements of evidence-based stroke care.

In terms of treatment, Australian data from the 2013 Stroke Foundation national acute audit found that only 45 % of patients with ischaemic stroke presenting to hospital within 3 h of stroke were assessed for tPA eligibility [8]. Only 7 % of eligible patients received tPA [8] with pockets of excellence where rates from individual sites were up to 21 % [9]. Less than optimal tPA rates also have been reported internationally; 12 % in the UK [10] and less than 5 % in the USA [11]. However, data from Norway, where collaboration for pre-hospital, ED and acute services is streamlined, demonstrate higher rates (31 %) are achievable [12]. In relation to the management of fever, hyperglycaemia and swallowing in Australia, pre-trial data from the 2013 Stroke Foundation national acute audit showed that only 60 % of patients received temperature monitoring four times a day during the first 72 h of admission, with only 36 % of those with a fever (>37.5 °C) receiving paracetamol within 1 h [8]. Less than a quarter (21 %) received four times a day glucose monitoring in the first 72 h of admission, and only 25 % patients with hyperglycaemia (blood glucose >10 mmol/L) received insulin within 1 h [8]. Two thirds (66 %) of patients received a swallowing screen or assessment within 24 h of admission [8], and of concern, only 52 % received a swallow screen/assessment prior to oral intake [8]. Our own Quality in Acute Stroke Care (QASC) trial data from the intervention group showed that 18 % of patients with stroke were given oral fluid or food before screening and 37 % were given oral medications [13]. Similarly, at the T^3^ Trial commencement, variable practices were reported regarding prompt transfer from ED to stroke units with ED length of stays ranging from a median of 7 h (maximum 20 h) [13] up to 11 h [14].

In summary, EDs must deliver time-critical, best-practice clinical care to optimise outcomes for patients with stroke. A specific challenge for EDs is the delivery of optimal care for patients with stroke whilst managing other patients with a range of illnesses and injuries of varying degrees of clinical urgency. It is clear that EDs need greater support to deliver evidence-based triage, treatment and transfer for patients presenting with acute stroke in order to improve patient outcomes. Building on our previous trial results [15], we aim to rigorously evaluate, using a cluster randomised controlled trial design, the effectiveness of a theory-informed, nurse-initiated, organisational intervention to improve multidisciplinary care for patients with acute stroke in EDs measuring outcomes at 90 days.

## Methods

### Hypothesis

#### Patient outcomes and quality of care

Compared to patients who receive care in EDs randomised to the control group, patients who receive care in EDs randomised to receive the T^3^ intervention will have:

- Patient primary outcome
  1. 10 % decrease in the proportion of patients dead or dependent 90 days post hospital admission (dependency defined as modified Rankin Score (mRS) ≥2)
- Patient secondary outcome
  2) 10 % increase in the proportion of patients with improved functional dependency 90 days post hospital admission (Barthel Index (BI) ≥95)
  3) 0.2 standard deviations higher mean SF-36 Mental Component Score (MCS) and Physical Component Score (PCS) 90 days post hospital admission (3.5 units for MCS; 2.5 units for PCS)
- Quality of care (in-hospital) secondary outcomes
  4) 15 % increase in the proportion of patients triaged to Australasian Triage Scale (ATS) category 1 or 2
  5) 20 % increase in the proportion of patients receiving assessment for tPA eligibility
  6) 10 % increase in the proportion of patients with temperature readings on admission and 4 hourly whilst in ED
  7) 10 % increase in the proportion of patients with a formal venous blood glucose level (BGL) sent to the laboratory on admission to ED
  8) 10 % increase in the proportion of patients with finger prick glucose readings on admission and at least 6 hourly whilst in ED
  9) 10 % increase in the proportion of patients who are either ‘Nil by Mouth’ or receive a swallow screen or assessment within 24 h of ED admission
  10) 10 % increase in the proportion of patients who remained ‘Nil by Mouth’ until they received a swallow screen or assessment
  11) 10 % increase in the patients who received a swallow screen or assessment within 24 h of ED admission
  12) 10 % decrease in the proportion of patients given oral fluids or food prior to a swallow screen or a swallow assessment
  13) 10 % decrease in the proportion of patients given oral medications prior to a swallow screen or a swallow assessment
  14) 10 % increase in the proportion of patients getting to the stroke unit within 4 h of presentation to the ED

### Study design

A prospective, multicentre, parallel group, blinded, cluster randomised controlled trial (CRCT) with blinded outcome assessment will be undertaken. The unit of randomisation will be EDs in order to minimise contamination as our intervention is designed for delivery at the organisational level addressing environmental or ‘systems’ impediments to best-practice stroke care in EDs. Outcomes will be assessed at the patient level.

### Eligibility and recruitment

#### Emergency departments

EDs at hospitals in three Australian states (NSW, VIC, QLD) and the Australian Capital Territory (ACT) with pre-existing dedicated stroke units will be eligible to participate. EDs at hospitals already participating in a stroke cluster randomised trial testing a thrombolysis intervention will be ineligible to participate to prevent possible contamination to either trial. We will meet ED Directors, ED Nurse Unit Managers (NUMs), ED Nurse Educators and the relevant Director of Allied Health as well as the Director of each hospital stroke unit and the stroke unit co-ordinator (or equivalent), to explain the aims of the trial. The ED Director will provide cluster guardian consent for the ED to be involved.

### Patients

Two patient cohorts will be recruited prospectively using identical methods by Clinical Research Assistants at each site. The first cohort will be recruited pre-intervention from all hospitals to provide baseline observational data. Data will be obtained from a consecutive sample of patients who are English-speaking, aged >18 years, have been admitted to the stroke unit via ED with a clinical diagnosis of ischaemic stroke or intracerebral haemorrhage and presented to hospital less than 48 h from symptom onset. Patients presenting later than this are unlikely to benefit from changes in clinical care and will be excluded. Also excluded will be those requiring palliative care only, with identified non-cerebrovascular causes of acute focal neurological deficits (seizure, hypoglycaemia, toxic or metabolic encephalopathies), sub-arachnoid haemorrhage, and acute and chronic subdural haemorrhage.

Demographic data (age, sex, stroke sub-type [scale explained below]) and stroke severity (scale explained below) will be obtained for all eligible consenting patients and also for eligible non-consenting patients to assess for selection bias. All eligible patients will be given a patient information statement outlining the study purpose, data collection and the 90-day follow-up process and information on how to opt-out/withdraw from the data collection. Patients in the post-intervention cohort will consent using an ‘opt-out’ approach. Consenting patients (or their friend/relative) will be asked to agree to be contacted by the researchers at 90 days to conduct a computer-assisted telephone interview (CATI) and to access their medical records. Patients will be approached in the stroke unit. Eligible patients missed whilst an in-patient on the stroke unit will be mailed the patient information statement. Patients may withdraw at any time without providing a reason. Recruitment rates by sites will be monitored by the Trial Manager (SD); no incentives for recruitment will be provided.

### Randomisation and allocation concealment

Hospitals will be randomised within strata defined by state and a baseline tPA rate (<7.7 vs >7.7 %; 7.7 % is the average annual rate based on the 2013 Stroke Foundation clinical audit [8]) in a 1:1 ratio to either intervention or control group. De-identified hospital and stratification details will be provided to a blinded statistician not otherwise involved in the trial to perform the randomisation using SAS Proc Plan and allocate EDs to their groups. Group allocation will be concealed until provided to the Trial Manager (SD).

### T^3^ intervention

#### Control group

EDs randomised to the control group will receive no additional support from the T^3^ Trialists nor receive any of the T^3^ Trial clinical protocols.

#### Intervention group

This organisational intervention will be implemented in EDs and will target healthcare professional behaviour for acute stroke management. The intervention is comprised of two components: the T^3^ protocols and the T^3^ implementation strategy, both described below.

#### The T^3^ protocols

In line with clinical practice guidelines, we have designed an evidence-based care bundle of clinical protocols for triage, treatment and transfer following acute stroke comprised of 12 clinical care elements (the T^3^ protocols). Developed by clinical experts, the T^3^ protocols will be delivered by nurses (i.e. nurse-initiated).

In order to prevent contamination before trial completion, we outline below broad components of our intervention, rather than provide details of each clinical behaviour as these will be published with the final trial results:

- *Triage*
  - Appropriate triage allocation by nurses for patients with suspected acute stroke
- *Treatment*
  - Assessment for eligibility for tPA
  - Administration of tPA to eligible patients
  - Fever, Sugar, Swallowing (FeSS) management. Whilst similar to those successfully used in the QASC trial [15] conducted by the researchers, updated versions of the FeSS clinical protocols, where required, will be developed by a multidisciplinary panel of experts
- *Transfer*
  - Prompt transfer of patients with stroke from ED to a stroke unit

### The T^3^ implementation strategy

#### Implementation intervention development

The science of implementation research is still evolving [16] with an incomplete but emerging evidence base to guide researchers and clinician leaders in the development of implementation interventions designed to implement practice change. The use of theoretical frameworks can assist with the systematic development of interventions for implementation trials [17, 18]. Acknowledging, however, that ‘no framework can address the level of detail required to determine what will or will not be an effective intervention’ [17, pg 3], we also retained elements of our previously successful implementation strategy used in the QASC trial [15, 19] consisting of workshops to determine barrier identification with local solutions; use of mixed interactive and didactic education; and use of local clinical opinion leaders and reminders (email, telephone and site visits).

The Theoretical Domains Framework (TDF) [1, 2] was chosen to further guide intervention design because we wished to comprehensively analyse the nature of the behaviours we were seeking to change and link them to specific behavior change techniques [17]. The TDF consists of 14 theoretical domains derived from 33 behaviour change theories, developed using a process of expert consensus with subsequent validation work [20]. It has been used elsewhere in healthcare settings to study implementation and more specifically assist the development of implementation interventions [21–23].

### Implementation strategy

The T^3^ protocols will be implemented using a theoretically informed [1, 2] and evidence-based behaviour change implementation strategy [15] consisting of workshops to determine barrier identification with local solutions [24]; use of mixed interactive and didactic education [25, 26]; use of local clinical opinion leaders (site clinical champions) [24]; reminders in the form of posters in ED, lanyard cards with listing the clinical care elements [27]; and sustained site engagement using site visits, telephone and email as described below:

- *Multidisciplinary workshops for ED and stroke unit clinicians (medical practitioners, nurses and speech pathologists) and endocrine clinicians* will be conducted by investigator nurses (SM and SD) with emergency, neurology and endocrine physician investigator researchers in attendance. Two face-to-face 60-min workshops will be held in the ED, 10 to 16 weeks apart with the same attendees. The first will target identification of local barriers, solutions and enablers to implementation of the T^3^ clinical care elements and also to reinforce enhanced team function. Any local adaptation to the clinical care elements required will be identified a priori. After the first workshop, site-specific action plans will be developed and discussed at the second workshop.
- *Interactive and didactic education program* for ED and stroke unit clinicians will be conducted explaining the intervention. A 15-min PowerPoint presentation will be delivered by Project Manager (SD) and T^3^ State Co-ordinators in one education session to ED and stroke unit clinicians collectively. The PowerPoint presentation and an 8-min video featuring an academic ED nurse (JC) explaining the 12 clinical care elements and their rationales will be provided to the site champions to deliver to any other members of the ED team and for ongoing education of new staff. T^3^ researchers will be available for any additional education sessions requested by individual sites if required.
- *Local clinical opinion leaders* (site clinical champions) from both the ED and the stroke unit identified at the first workshop (described above) will drive clinical change locally.
- *Reminders* will be aimed at sustained engagement of ED and stroke unit champions*.* This will consist of posters placed in the ED by site champions and lanyard cards listing the 12 clinical care elements.
- *Sustained engagement* of ED and stroke unit champions to embed organisational linkages and collaboration by the T^3^ state co-ordinators by 6-weekly site contact to discuss progress against the action plans, alternating between site visits and teleconferences; and follow-up of reactive telephone calls and emails initiated by local site champions.

Our intervention is aimed at behaviour change at both the individual clinician level and the organisational level.

### Implementation fidelity

In order to maintain fidelity of our intervention delivery, all workshop and education sessions will be facilitated by one or more of the researchers (SM, SD, LC, VS); all of whom will undergo training to promote consistency in delivery methods and content. Workshops will be audiotaped to enable data collection of barriers and ensure the development of accurate and relevant action plans. A standardised PowerPoint presentation will ensure the didactic component of the education will be consistently delivered.

### Outcome measures

#### Ninety-day patient outcomes: telephone interview (intervention outcomes)

Patients will be telephoned 90 days post-admission and asked to complete a 30-min computer-assisted telephone interview (see data collection procedure below) using previously validated and commonly used scales to measure:

- *Death or dependency:* modified Rankin Score (mRS) of ≥2 (primary outcome). The mRs is a 6-point scale where 0 = independent and 6 = dead [28].
- *Functional dependency*: Barthel Index (BI). The BI measures patient performance in 10 activities of daily life relating to self-care (feeding, grooming, bathing, dressing, bowel/bladder care, toilet use) and mobility (ambulation, transfers, stair climbing) [29].
- *Health status:* Medical Outcomes Study Short Form 36 Health Survey Questionnaire (SF-36). The SF-36 includes a single ‘health transition rating’ and scores eight health domains which are aggregated to form the Physical Component Score (PCS) and the Mental Component Score (MCS) [30].
- *Health-related quality of life:* EuroQol 5D (EQ-5D) measures five dimensions of care: mobility, self-care, usual activities, pain and discomfort, and anxiety and depression, and will inform the economic evaluation [30].

### Clinical data and quality of care outcome measures—retrospective medical record audits (implementation outcomes)

The following clinical measures and patient characteristics will be collected from patient medical records: age; sex; pre-morbid mRS; length of hospital stay; time from onset of symptoms to arrival in ED; stroke sub-type using the Oxfordshire Community Stroke Project (OCSP) Classification [31] (a four-item scale that classifies strokes using explicit criteria as either lacunar infarcts, total anterior circulation infarcts, partial anterior circulation infarcts or posterior circulation infarcts); and stroke severity using the National Institutes of Health Stroke Scale (NIHSS) score [32] (ranges from 0 to 42, with higher values reflecting more severe cerebral infarcts); diabetes status; and stroke risk factors (past history of stroke/TIA, hypertension, hyperlipidaemia, smoking status).

We also will collect data to measure the quality of care outcomes and implementation efficacy as follows: ATS category allocation; time to first seen by an ED doctor; assessment of eligibility for tPA and result (eligible/not eligible); receipt of tPA; all temperature and blood glucose measurement times and values whilst in ED and in stroke unit up to 72 h following admission; paracetamol and insulin administration and mode of delivery; swallow screening data (whether a swallow screen was performed in ED, and before patients were given food, drink or medications); whether those who fail the screen were reviewed by a speech pathologist; and length of time spent in the ED and the stroke unit.

### Data collection process, data entry and data storage

Patients will be contacted by telephone 90 days following admission by the computer-assisted telephone interview (CATI) research assistant (RA) blind to the study design and group allocation. The CATI RA will undergo training in telephone administration of the study measures and mRS assessment certification and will enter all 90-day data into the database. One week prior to the CATI, a reminder letter will be mailed to patients as a response-aiding strategy. This also enables relatives of any deceased patients to contact us to inform us of their family member’s demise. Where patients’ level of disability or dysphasia precludes them from talking on the telephone, a relative or carer will be invited to respond on behalf of the patient as a proxy.

Independent research assistants not otherwise involved in the study, blind to the group allocation and study design, will be trained to undertake the retrospective medical record audits and data entry. Interrater reliability will be established by independent double-auditing 10 % of randomly selected records, based on computer-generated random numbers, with a minimum of five records per site for the pre- and post-intervention audit, respectively. Agreement between auditors will be assessed using Kappa statistic [33].

### Implementation of the intervention

After a 3-month bedding down period [15] to allow our intervention to lead to behaviour change that has become routine care, the second ‘post-intervention’ cohort of patients will be recruited prospectively from all hospitals to provide post-intervention outcome data using similar tools and methods to those used to collect pre-intervention data.

### Blinding

Patients will be blinded to the trial aim and group allocation. Research assistants collecting the 90-day outcome measures and those collecting the medical record audit data will be masked to trial aims, design and group allocation; the trial statisticians will be blinded to the group allocation. Clinicians delivering the intervention in EDs and clinicians in their respective stroke units will not be blinded to the group allocation.

### Sample size

We plan to recruit 1160 patients following implementation of the intervention in our post-intervention cohort, anticipating a 10 % loss to follow-up [15]. Our trial will have 80 % power at a 5 % significance level with the following assumptions based on our previous QASC trial: 50 % of patients in the usual care group will have mRS ≥2, 60 % will have Barthel Index ≥95, standard deviations for MCS and PCS of 11 and a design effect of 1.4. This will allow detection of a difference between intervention and control groups at 90 days post-admission of 10 % for mRS ≥2 (primary outcome); 10 % for Barthel Index (BI)) ≥95 (secondary outcome 1); and 0.2 standard deviations (approximately 2.5 units) for SF-36 Physical Component Score (PCS) (secondary outcome 2) and SF-36 Mental Component Score ([MCS) (secondary outcome 3). Similarly, we will be able to detect a difference in the process of care outcomes between intervention and control groups of at least 10 %.

### Statistical methods

An intention-to-treat analysis will be undertaken using the Stata statistical package. Demographic and clinical characteristics will be presented by group.

Analysis of outcomes will involve regression (logistic or linear as appropriate) with adjustment for baseline values of the outcome and correlation of outcomes within hospitals. We will undertake unadjusted analyses, as well as analyses which adjust for baseline covariates. Primary analysis will be a complete case analysis with sensitivity analyses using multiple imputations to account for missing data, ensuring that all patients are included in an intention-to-treat analysis.

Adherence to specific protocol analysis will be undertaken using the 12 clinical care elements or variations agreed a priori with each individual hospital. A planned economic evaluation also will be conducted for which a separate protocol will be published. No interim analyses are planned.

### Examination of contextual factors influencing knowledge transfer: a process evaluation

At the conclusion of the T^3^ Trial, we will conduct focus groups and semi-structured interviews to examine the contributing organisational, contextual and structural factors that may explain successful uptake of the T^3^ intervention.

All data will be stored, managed and archived in accordance with National Health and Medical Research Council requirements. Data transferred to third parties will be password-protected. We will archive the final trial data set in a data repository. Only de-identified data will be analysed. Data transcripts from focus groups and interviews will not be identified by participant name, but an identification number will link a participant’s name in a file which will be stored separately from the transcripts. No identified data will be published or released. All study material will be disposed of in a confidential manner by shredding all interviews transcripts and erasing all audiotapes and computer files. The following authors have access to the full data sets SM, SD and EM; CDE and PM will have access to the de-identified data sets. Manuscripts will be prepared for peer-reviewed publication to communicate trial results regardless of the magnitude or direction of effect.

**Fig. 1:**
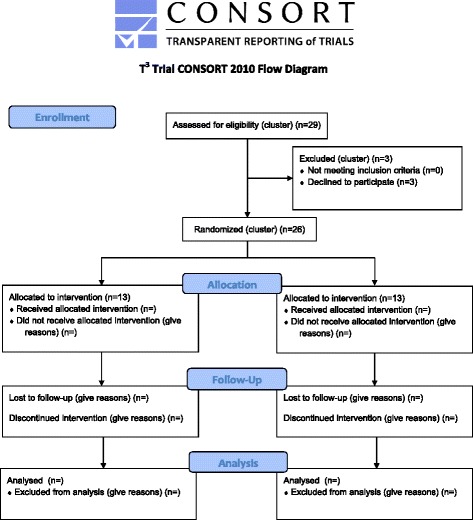
CONSORT flow diagram of the progress through the T^3^ Trial

At time of publication, EDs have been randomised and patients are being recruited, with the first patient recruited in July 2015 (Fig. 1). Further details about the T^3^ Trial enrolment,  interventions and assessments are shown in the SPIRIT flow diagram (Additional file 1).

## Discussion

There are approximately 60,000 new or recurrent strokes annually in Australia [34]. Cost of subsequent lifetime care has been estimated to exceed $2 billion [35]. Whilst extensive research has confirmed the importance of in-patient stroke unit care as a positive predictor of survival and recovery [36], it is expected that even better results will be realised with focused evidence-based stroke care in EDs before transfer to a dedicated stroke unit. Acknowledging the complexity of the ED environment [37], our trial uses an evidence-based, multifaceted and multidisciplinary approach to address key elements of ED stroke care with demonstrated evidence-practice gaps.

We have built on our previously successful QASC trial results by replicating and enhancing our proven intervention using a theoretical framework, the Theoretical Domains Framework to guide the refinement of our T^3^ intervention aiming to maximise intervention uptake by clinicians. A strength of our trial is that we are undertaking a process evaluation alongside our CRCT [38].

Stroke is common and costs large if not treated according to evidence-based guidelines during all phases of hospital admission. To improve the ‘whole pathway’ in stroke, care between EDs and stroke units must be more collaborative and evidence-based. Nurses are well placed to lead this collaborative in-patient care [39]. Community interest in quality of care, healthcare efficiency and post-discharge outcomes is increasing [40]. We believe the study’s rigour, timeliness and novelty will also set new benchmarks for hospital-based implementation research internationally.

## Additional file

Additional file 1: SPIRIT Flow diagram of the progress through the T^3^ Trial: schedule of enrolment, interventions, and assessments. (DOC 41 kb)

## Abbreviations

ACT: Australian Capital Territory
ATS: Australasian Triage Scale
BI: Barthel Index
CATI: Computer-assisted telephone interview
CRCT: Cluster randomised controlled trial
EDs: Emergency departments
EQ-5D: EuroQol 5D
HREC: Human Research Ethics Committee
MCS: Mental Component Score
mRS: Modified Rankin Score
NIHSS: National Institutes of Health Stroke Scale
NSW: New South Wales
NUM: Nurse Unit Managers
OCSP: Oxfordshire Community Stroke Project
PCS: Physical Component Score
QASC: Quality in Acute Stroke Care
QLD: Queensland
RA: Research assistant
T^3^: Triage, treatment and transfer
TDF: Theoretical Domains Framework
TIA: Transient ischaemic attack
tPA: Tissue plasminogen activator
VIC: Victoria
