# Supplementary material for: Triage, treatment and transfer of patients with stroke in emergency department trial (the T3 Trial): a cluster randomised trial protocol
Source: Implement Sci. 2016 Oct 18;11:139. doi: 10.1186/s13012-016-0503-6 (PMC5069775; doi:10.1186/s13012-016-0503-6)
Supplement: Supplementary file 1 [file 13012_2016_503_MOESM1_ESM.doc]

T^3 Trial: schedule of enrolment, interventions, and assessments

	STUDY PERIOD							
		Pre-intervention cohort		Randomisation	Post-Intervention cohort		End of patient recruitment	Close-out/ Completion of trial
**Dates**	***2012***	**July 2015-June 2014**		**Jul-15**	***July 2015-Oct 2016***		***Oct-16***	***Dec-17***
**TIMEPOINT****	***On hospital enrolment***	***On patient enrolment***	***90-day post patient admission***		***On patient enrolment***	***90-day post patient admission***		
ENROLMENT:								
Eligibility screen Hospitals	X							
Informed consent Hospitals	X							
Eligibility screen Patients		X			X			
Informed Consent Patients		X			X			
Randomisation of hospitals				X				
INTERVENTIONS:								
T^3 Intervention (Triage, treatment & transfer) clinical protocols + implementation				X	———	———	X	
ASSESSMENTS:								
Pre-morbid mRS		X			X			
Stroke sub-type (OCSP classification)		X			X			
Stroke severity (NIHSS score)		X			X			
PATIENT OUTCOMES & QUALITY OF CARE								
Death and dependency (mRS)			X			X		
Health status (SF-36)			X			X		
Functional dependency (Barthel Index)			X			X		
Quality of life (EQ-5D)			X			X		
Quality of care in hospital secondary outcomes (medical record audits)		X	———	———	———	———	X	
Process analysis								X

*Recommended content can be displayed using various schematic formats. See SPIRIT 2013 Explanation and Elaboration for examples from protocols.

**List specific timepoints in this row.
